# Integrated clustering of multiple immune marker trajectories reveals different immunotypes in severely injured patients

**DOI:** 10.1186/s13054-024-04990-4

**Published:** 2024-07-15

**Authors:** Maxime Bodinier, Estelle Peronnet, Jean-François Llitjos, Louis Kreitmann, Karen Brengel-Pesce, Thomas Rimmelé, Aurore Fleurie, Julien Textoris, Fabienne Venet, Delphine Maucort-Boulch, Guillaume Monneret, REALISM study group, Sophie Arnal, Caroline Augris-Mathieu, Frédérique Bayle, Liana Caruso, Charles-Eric Ber, Asma Ben-Amor, Anne-Sophie Bellocq, Farida Benatir, Anne Bertin-Maghit, Marc Bertin-Maghit, André Boibieux, Yves Bouffard, Jean-Christophe Cejka, Valérie Cerro, Jullien Crozon-Clauzel, Julien Davidson, Sophie Debord-Peguet, Benjamin Delwarde, Robert Deleat-Besson, Claire Delsuc, Bertrand Devigne, Laure Fayolle-Pivot, Alexandre Faure, Bernard Floccard, Julie Gatel, Charline Genin, Thibaut Girardot, Arnaud Gregoire, Baptiste Hengy, Laetitia Huriaux, Catherine Jadaud, Alain Lepape, Véronique Leray, Anne-Claire Lukaszewicz, Guillaume Marcotte, Olivier Martin, Marie Matray, Delphine Maucort-Boulch, Pascal Meuret, Céline Monard, Florent Moriceau, Guillaume Monneret, Nathalie Panel, Najia Rahali, Thomas Rimmele, Cyrille Truc, Thomas Uberti, Hélène Vallin, Fabienne Venet, Sylvie Tissot, Abbès Zadam, Sophie Blein, Karen Brengel-Pesce, Elisabeth Cerrato, Valérie Cheynet, Emmanuelle Gallet-Gorius, Audrey Guichard, Camille Jourdan, Natacha Koenig, François Mallet, Boris Meunier, Virginie Moucadel, Marine Mommert, Guy Oriol, Alexandre Pachot, Estelle Peronnet, Claire Schrevel, Olivier Tabone, Julien Textoris, Javier Yugueros Marcos, Jérémie Becker, Frédéric Bequet, Yacine Bounab, Florian Brajon, Bertrand Canard, Muriel Collus, Nathalie Garcon, Irène Gorse, Cyril Guyard, Fabien Lavocat, Philippe Leissner, Karen Louis, Maxime Mistretta, Jeanne Moriniere, Yoann Mouscaz, Laura Noailles, Magali Perret, Frédéric Reynier, Cindy Riffaud, Mary-Luz Rol, Nicolas Sapay, Trang Tran, Christophe Vedrine, Christophe Carre, Pierre Cortez, Aymeric de Monfort, Karine Florin, Laurent Fraisse, Isabelle Fugier, Sandrine PAYRARD, Annick Peleraux, Laurence Quemeneur, Andrew Griffiths, Stephanie Toetsch, Teri Ashton, Peter J. Gough, Scott B. Berger, David Gardiner, Iain Gillespie, Aidan Macnamara, Aparna Raychaudhuri, Rob Smylie, Lionel Tan, Craig Tipple

**Affiliations:** 1 EA 7426 “Pathophysiology of Injury-Induced Immunosuppression” (Université Claude Bernard Lyon 1 - Hospices Civils de Lyon - bioMérieux), Joint Research Unit HCL-bioMérieux, Immunology Laboratory and Anesthesia and Critical Care Medicine Department, Hospices Civils de Lyon, Edouard Herriot Hospital, 5 place d’Arsonval, 69003 Lyon Cedex 03, France; 2 Department of Infectious Disease, Faculty of Medicine, Imperial College, London, W12 0NN UK; 3 Anesthesiology and Critical Care Medicine, Edouard Herriot Hospital, Hospices Civils de Lyon, 69003 Lyon, France; 4 Centre International de Recherche en Infectiologie (CIRI), Inserm U1111, CNRS, UMR5308, Ecole Normale Supérieure de Lyon, Université Claude, Bernard-Lyon 1, Lyon, France; 5 Université Claude Bernard Lyon 1, Université de Lyon, Lyon, France; 6 Équipe Biostatistique-Santé, Laboratoire de Biométrie Et Biologie Évolutive, CNRS UMR 5558, Villeurbanne, France; 7 Service de Biostatistique-Bioinformatique, Pôle Santé Publique, Hospices Civils de Lyon, Lyon, France

**Keywords:** Sepsis, Critical illness, Immune markers, Trajectory, Longitudinal study, Patient stratification, Immune response, Transcriptomic, Immunosuppression

## Abstract

**Background:**

The immune response of critically ill patients, such as those with sepsis, severe trauma, or major surgery, is heterogeneous and dynamic, but its characterization and impact on outcomes are poorly understood. Until now, the primary challenge in advancing our understanding of the disease has been to concurrently address both multiparametric and temporal aspects.

**Methods:**

We used a clustering method to identify distinct groups of patients, based on various immune marker trajectories during the first week after admission to ICU. In 339 severely injured patients, we initially longitudinally clustered common biomarkers (both soluble and cellular parameters), whose variations are well-established during the immunosuppressive phase of sepsis. We then applied this multi-trajectory clustering using markers composed of whole blood immune-related mRNA.

**Results:**

We found that both sets of markers revealed two immunotypes, one of which was associated with worse outcomes, such as increased risk of hospital-acquired infection and mortality, and prolonged hospital stays. This immunotype showed signs of both hyperinflammation and immunosuppression, which persisted over time.

**Conclusion:**

Our study suggest that the immune system of critically ill patients can be characterized by two distinct longitudinal immunotypes, one of which included patients with a persistently dysregulated and impaired immune response. This work confirms the relevance of such methodology to stratify patients and pave the way for further studies using markers indicative of potential immunomodulatory drug targets.

**Graphical Abstract:**

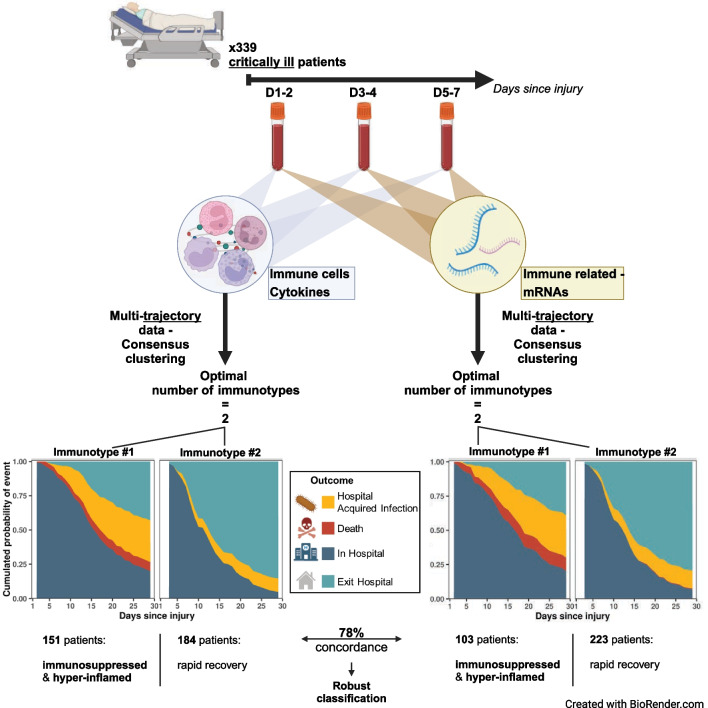

**Supplementary Information:**

The online version contains supplementary material available at 10.1186/s13054-024-04990-4.

## Introduction

Sepsis is an important public health concern [1] that can result in potentially fatal organ dysfunction due to a dysregulated host response to infection [2]. This dysregulation primarily affects the immune system, leading to a complex interplay between pro- and anti-inflammatory pathways that influence disease progression and clinical outcomes such as organ dysfunction, the occurrence of hospital-acquired infections, and mortality [3]. However, attempts to treat sepsis patients with immune therapies have had limited success due to the intricate and heterogeneous nature of the immune response in sepsis [4].

Most studies investigating the heterogeneity of the immune response in sepsis have utilized a single time-point, often just after ICU admission, to characterize the host response [5–8]. However, given the dynamic nature of the immune response in sepsis, longitudinal studies are necessary to fully understand its heterogeneity [3, 9, 10]. While there have been several studies exploring the longitudinal immune response in sepsis, they have focused on single-marker approaches, thus limiting our understanding of the complex nature of this response [11–13]. In parallel, recent research has shown that post-immune injury responses are shared across various critical illness conditions such as severe trauma and major surgery [10, 14]. These elements underscore the importance of an integrated approach to characterize the heterogeneity of immune responses, also known as phenotyping [15], which can ultimately inform personalized treatment strategies, as proposed in the literature through the concept of prognostic enrichment and treatable traits [16–18].

The aim of this study was to decipher the dynamic longitudinal heterogeneity of the immune response during the first week following admission of critically ill patients by identifying distinct groups of patients via a multi-marker clustering approach. We used the term "immunotype" to describe groups of patients that exhibit similar immune responses to the initial injury. To accomplish this, we utilized two sets of immune markers: a set of immune markers commonly described in literature and a set of whole blood immune related mRNA markers. We also investigated the association between these immunotypes and deleterious outcomes such as nosocomial infection, length of stay, and mortality.

## Materials and methods

### Patient cohort

For immune longitudinal characterization, we analyzed a total of 353 critically ill patients from the REALISM cohort [14] which included 107 septic patients, 137 trauma patients and 109 patients who underwent major surgery. This cohort recruited patients admitted to Lyon University Hospital in France between December 2015 and June 2018. The REALISM study design has been thoroughly discussed in Rol et al. [19] and in Venet et al. [14]. The study design excluded patients under immunosuppressive treatment before admission. The REALISM cohort is registered on ClinicalTrials.gov (NCT02638779) and was approved by the Institutional Study Board (2015-42-2). Additionally, the REALISM cohort comprised 175 healthy volunteers, representative of the age and sex distribution in the French population in 2016. These volunteers were aged between 18 and 82 years, with 81 males and 94 females.

Blood samples were collected from each patient on either Day 1 or 2 of admission to the critical care unit. Additional blood samples were collected on Days 3 or 4, on Days 5, 6, or 7. These specific sampling days were chosen to capture the early and late phases of critical illness when immunological changes are expected to occur and were used to assess longitudinal immunotypes. When available, Day 14 sample was used to assess immune recovery of identified first week immunotypes.

### Immune markers measurement

In the REALISM cohort study, two sets of markers were analyzed (see study protocol [19]): the Reference marker set (REF set) and the mRNA marker set (mRNA set). Markers were controlled for colinearity below 0.8 (see Supplementary Figs. S1 and S2) to ensure unique, independent information from each marker. The REF set consisted of known biomarkers of sepsis immunosuppression [20]: percentage of immature neutrophils, monocytic HLA-DR expression, T cell counts, and the interleukin (IL) 6 and IL-10 plasma concentrations. The mRNA set consisted of 5 markers selected based on their involvement in selected immune function: CD74 (involved in antigen presentation), CX3CR1 (chemokine receptor involved in immune cell recruitment and activation), IL7R (important for T cell development and survival), IFNg (pro-inflammatory cytokine) and IL1R2 (decoy receptor for the pro-inflammatory cytokine interleukin-1). They were analyzed through a multiplexed RT-qPCR platform: the FilmArray Torch Instrument (BioFire) with the IPP prototype as previously described in [21].

To assess immune recovery, Immune functional assays were measured following protocole previously described in [22, 23].

### Outcomes

The primary clinical outcome of our study was the composite outcome “Complicated Hospital Course” (CHC), which is defined as the occurrence of either healthcare-associated infections—HAI or death within 30 days or an ICU stay longer than 7 days. HAI monitoring began 72 h after the initial injury, with all HAI episodes reviewed and validated by a blinded adjudication committee consisting of three physicians.

Secondary clinical outcomes included the occurrence of HAI within 30 days, all-cause 30-day and 90-day mortality, and the ICU-free days at day 30, hospital-free days at day 30, and mechanical ventilation-free days at Day 30. These outcomes were monitored and documented throughout the study.

### Statistical analysis

An overview of the study workflow is represented in Supplementary Fig. S3.

#### Defining immunotypes through unsupervised clustering

In this study, we employed an unsupervised clustering method to classify patients into distinct groups, termed as “immunotypes”, based on the similarities observed in their immune response trajectories. These immunotypes essentially represent a cohort of patients whose immune responses demonstrated analogous patterns over a period of time. We utilized a flexible, non-parametric method known as KmL-3D for the purpose of clustering these trajectories, taking into account their co-evolution [24]. The selection of this method was primarily due to its capability to handle missing data and accommodate multiple trajectories. The final count of immunotypes was empirically determined, relying on the stability of patient grouping within a consensus clustering framework. This method essentially mitigates the risk of overfitting by summarizing the stability of clustering (PAC—Proportion of ambiguous clusters metric) based on the application of KmL3D on 100 bootstrap iterations of the initial dataset [25]. Patient membership was derived by performing hierarchical clustering on the most stable consensus clustering matrix. Lastly, we employed a method known as LOESS to smooth marker measurements within each immunotype, thereby enabling us to define the mean evolution of each immunotype.

Further details pertaining to the methodology are provided in the Supplementary Methods, and a summary of the method pipeline can be found in Supplementary Fig. S4.

#### Clinical characterization

To describe the different patient groups in our study, we assessed several clinical variables including demographics, admission characteristics and outcomes. Continuous data were summarized by median, interquartile range. Categorical data were summarized by sample sizes and percentages. Clinical characteristics of immunotypes were compared either with the analysis of variance (ANOVA) test in case of normally distributed data or with the Kruskal Wallis test by ranks for continuous data. Chi-squared tests or Fisher’s exact tests were used for categorical data.

Normality of data distribution was assessed using Shapiro–Wilk tests. The null hypothesis was rejected for a p value less than 0.05. All statistical analysis and visualizations were done using R 4.1.3 [26]. All graphics were done using ggplot v3.3.2 [27].

#### Competing risk analysis

To analyze the incidence of HAI outcome occurrence in the REALISM cohort, we employed a competing risk framework, considering hospital discharge and death as competing events (all censored at day 30). We used the survival 3.5-5 and survminer 0.4.9 R packages for this analysis, computing cumulative incidence functions (CIFs) to estimate the incidence of HAI while taking into account competing events. For evaluating the impact of different immunotypes on these outcomes, we applied the Fine and Gray model (in the R package cmprsk 2.2-11), which extends the traditional Cox proportional hazards model to accommodate competing risks. We computed sub-distribution hazard ratios (sHR) and their 95% confidence intervals (CI) to assess the association between immunotypes and HAI, death, and hospital discharge.

## Results

### REALISM cohort characteristics

From the 353 patients initially included in the REALISM cohort, we excluded a total of 18 time points that occurred after HAI events, since an infection can modulate immune marker levels (Supplementary Fig. S5). We removed 14 patients with only one time point from the dataset, as we could not study biomarker trajectories for them. The final subcohort analyzed was composed of a total of 339 patients. For most cases (n = 241), we measured biomarker levels thrice a week, and the time points for the trajectories were evenly distributed over the first week, in three periods: D1 to D2, D3 to D4, and D5 to D7. Patient’s baseline characteristics and outcomes are presented in Supplementary Table S1 and have been described in more details in reference [16]. There were 151 patients (44%) who experienced the composite outcome CHC (Supplementary Table S2).

### Immunotypes definition

#### REF set biomarkers immunotypes

**Fig. 1:**
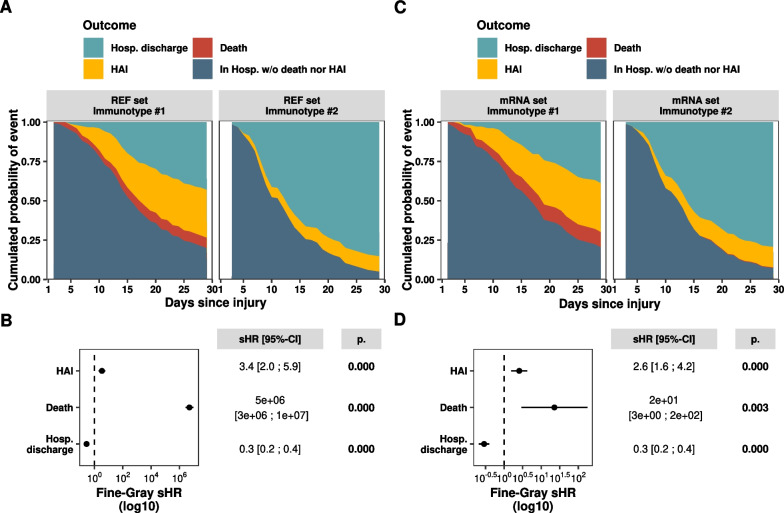
REF set and mRNA set immunotypes HAI incidence, with death and hospital discharge as competing risks. **A** For each of the two REF set trajectory immunotypes: cumulative incidence of HAI with death and hospital discharge as competing risks, Immunotype #1 (left) and Immunotype #2 (right) up to D30 follow-up. **B** Forest plot of Fine-Gray regression subdistribution Hazard Ratios (sHR) of outcomes, comparing REF set Immunotype #1 with Immunotype #2. sHR values are depicted graphically (black points) and numerically, along with 95% Confidence Intervals (CI, horizontal bars). sHR values significantly different from 1 are displayed in bold, and the corresponding *p* values (*p*.) are reported numerically. **C** For each of the two mRNA set trajectory immunotypes: cumulative incidence of HAI with death and Hospital discharge as competing risks in validation cohort predicted Immunotype #1 (left) and Immunotype #2 (right) up to D28 follow-up. **D** Forest plot of Fine-Gray regression subdistribution Hazard Ratios (sHR) of outcomes, comparing mRNA set Immunotype #1 with Immunotype #2. sHR values are depicted graphically (black points) and numerically, along with 95% Confidence Intervals (CI, horizontal bars). sHR values significantly different from 1 are displayed in bold, and the corresponding *p* values (*p*.) are reported numerically. HAI: Healthcare Associated Infection. REF set: plasmatic IL6, plasmatic IL10, HLA-DR antibody/monocyte, T cells blood concentration, and percentage of immature neutrophils. mRNA set: normalized mRNA Cp in whole blood, IFNG, CD74, CX3CR1, IL7R, and IL1R2

In the analysis of the five reference biomarkers (REF set), we included 335 patients out of the 339 who had trajectory data with at least two measurements over the first week for the five REF set markers (Supplementary Fig. S5). The PAC metric indicated that two immunotypes provided the most stable clustering of the trajectories (Supplementary Fig. S6A). The clinical characteristics (Table 1, Fig. 1A, B) of the resulting 2 immunotypes showed that Immunotype #1 (n = 151) was associated with poorer outcomes in comparison to Immunotype #2 (n = 184), with a higher proportion of patients experiencing a complicated hospital course (66% vs. 26%). All secondary endpoints also showed a significantly higher incidence in Immunotype #1 than in Immunotype #2, including higher incidence of 30-day HAI (32% vs. 11%, sHR [CI] 3.39 [1.96; 5.87]), 30-day mortality (9% vs. 0%, sHR [CI] 5.1e6 [2.7e6; 9.7e6]), ICU stays > 7 days (46% vs. 16%), and 90-day mortality (13% vs. 3%). These findings underscore the association between Immunotype #1 and adverse clinical outcomes. Moreover, these associations remained significant even after adjusting for clinical characteristics at admission, such as age, SOFA score, Charlson comorbidity index score, and initial injury, further emphasizing the interest of such clustering (Supplementary Table S3). We additionally checked if the observed immunotypes could be solely explained by initial characteristics, and found that the capacity of classification into immunotypes by those characteristics was moderate, with an AUROC of 0.78 [0.74–0.82] (Supplementary Fig. S7A).

**Table 1:** REF set immunotypes clinical characterization

	REF set Immunotype #1 (n = 151)	REF set Immunotype #2 (n = 184)	*p*. Value
Baseline characteristics			
Category at admission			
Sepsis/Septic shock	74 (49%)	25 (14%)	** < 0.001**
Trauma	46 (30%)	90 (49%)	
Surgery	31 (20%)	69 (38%)	
Female gender	45 (30%)	70 (38%)	0.143
Age, years	62 [49–72]	56 [44–70]	**0.013**
Body mass index, kg/m^2^	24 [22–27]	26 [22–30]	**0.017**
Charlson score	1 [0–3]	1 [0–2]	0.067
Parameters at admission			
SAPS II score	37 [26–49]	23 [16–33]	** < 0.001**
SOFA score	8 [4–10]	2 [1–5]	** < 0.001**
Mechanical ventilation	102 (68%)	53 (29%)	** < 0.001**
Vasopressor use	112 (74%)	57 (31%)	** < 0.001**
D1 Lymphocyte	1.03 [0.63–1.67]	1.25 [0.90–1.81]	**0.005**
D1 NLR	13.33 [8.42–21.04]	7.90 [5.31–11.57]	** < 0.001**
Treatments during first week			
Hydrocortisone Hemisuccinate	21 (13.9%)	4 (2.2%)	** < 0.001**
Outcomes			
CHC (Complicated Hosp. Course)	100 (66%)	48 (26%)	** < 0.001**
D30 HAI	49 (32%)	21 (11%)	** < 0.001**
D30 Death	13 (9%)	1 (0%)	** < 0.001**
ICU LOS > 7D	65 (46%)	23 (16%)	** < 0.001**
D90 Death	20 (13%)	6 (3%)	**0.002**
D30 ICU free days	21 [12–24]	26 [23–27]	** < 0.001**
D30 Hosp. free days	4 [0–15]	18 [12–22]	** < 0.001**
D30 Mech. Vent. free days	27 [21–29]	29 [27–29]	** < 0.001**

SAPS II: Simplified Acute Physiological Score II. SOFA: Sequential Organ Failure Assessment score. D: Day. MV: mechanical ventilation. ICU: Intensive Care Unit. HAI: Healthcare Associated Infection. REF set: plasmatic IL6, plasmatic IL10, monocytic HLA-DR antibody / cell, T cells blood concentration, and percentage of immature neutrophils. Data are presented as numbers and percentages (qualitative variables) and medians and 25th/75th percentiles (quantitative variables). Cohorts were compared either with analysis of variance (ANOVA) test in case of normally distributed data or with Kruskal Wallis test by ranks for continuous data, and Chi-squared test or Fisher’s exact test, where required, for categorical data. *p*. Values less than or equal to 0.05 were considered statistically significant and are highlighted in bold

**Fig. 2:**
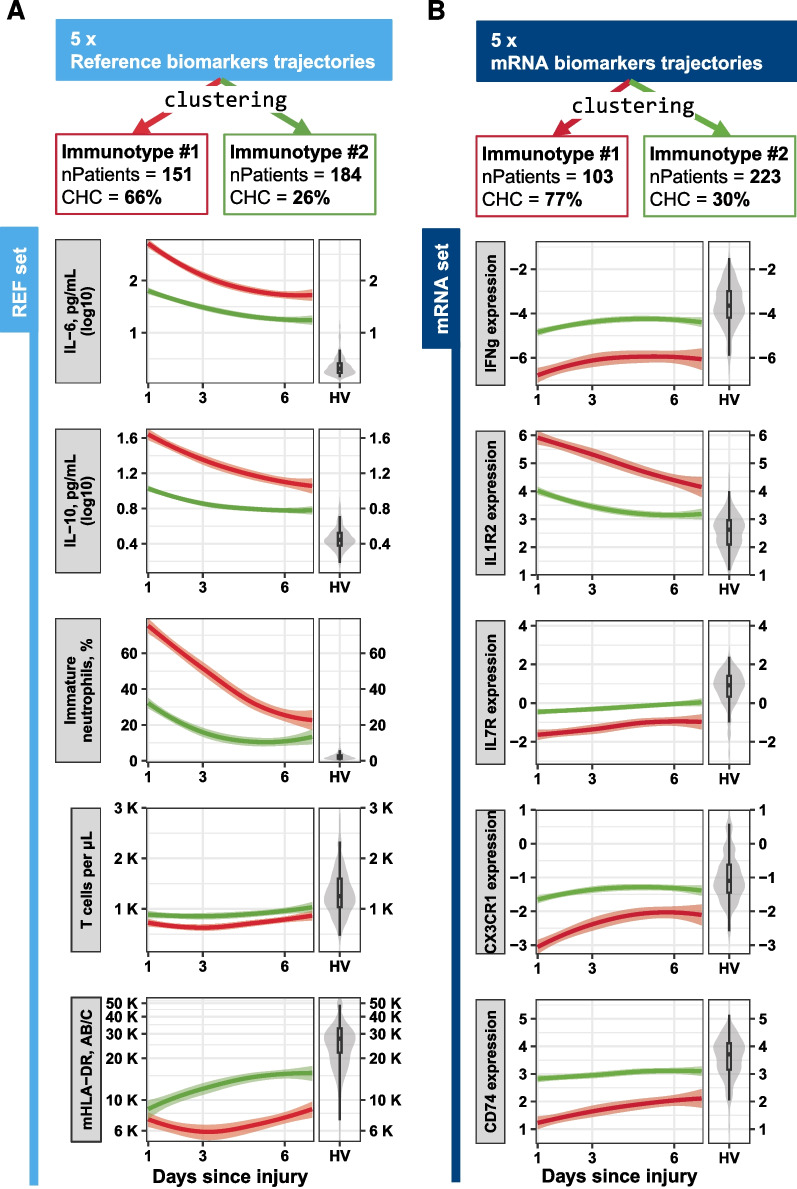
Longitudinal Immunotype characterization of critically ill patients using REF set and mRNA set markers. Critically ill patients (sepsis, severe trauma, and major surgery) were consecutively measured at D1-2, D3-4, and D5-7 after injury for 5 reference markers (REF set, **A**) and 5 mRNA markers (mRNA set, **B**). Trajectory clustering was performed for each set, with all 5 markers' temporal evolution considered together, exploring from 2 to 6 clusters. The resulting clusters are referred to as immunotypes and are represented as boxes on the top of the figure, with the first row indicating the immunotype label, the second row showing the number of patients (“nPatients”), and the third row displaying the enrichment in complicated hospital course (“CHC”), defined as the presence of one or more complications such as D30 Healthcare Associated Infection, more than 7 days in ICU, or D30 death. Temporal evolution of each of the 5 markers used for immunotypes construction were drawn below, with time in days after injury represented on x-axis, and marker level on y-axis. Immunotype #1 is represented in red, and Immunotype #2 in green. The evolution of markers is depicted through loess regression within each identified Immunotype, with standard error around mean curves. On the right side of each plot, the reference distribution of healthy volunteers (HV) is shown as violin plots for comparison. REF set: plasmatic IL6, plasmatic IL10, HLA-DR antibody per monocyte (mHLA-DR), T cells blood concentration per microliter, and percentage of immature neutrophils in blood. mRNA set: normalized mRNA Cp in whole blood, IFNg, CD74, CX3CR1, IL7R, and IL1R2

Examining the mean trajectories of the REF set biomarkers within each of the two identified immunotypes (Fig. 2), we found a notable divergence from healthy volunteers’ levels at the inclusion, converging towards these levels by the end of the first week. In comparison to Immunotype #2, Immunotype #1 exhibited a distinct immune profile characterized by elevated levels of IL6 and IL10, a substantial increase in immature neutrophils (peaking at 80% within the first week post-injury and declining to about 20% by the end of this period), and consistently low mHLA-DR levels (remaining below 8–10,000 AB/C throughout the week). These patterns suggest a more pronounced dysregulation of the immune system in Immunotype #1 during the immediate post-injury phase. Interestingly, T cell counts were comparable between the two immunotypes and appeared within the range observed in healthy volunteers.

**Fig. 3:**
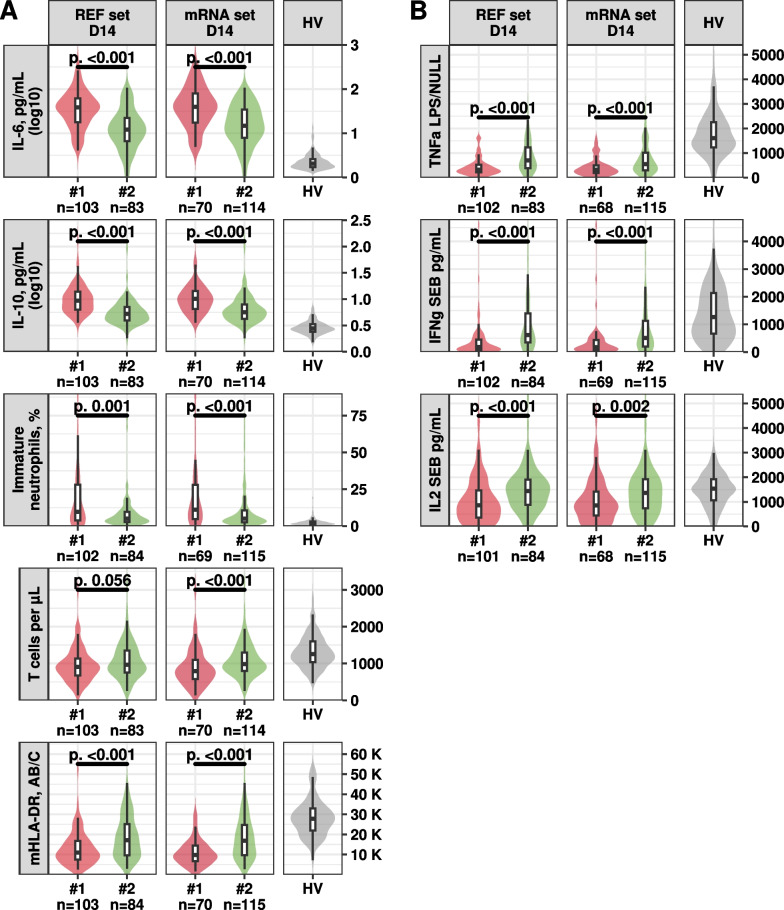
D14 immune system differences per REF set Immunotypes or mRNA set Immunotypes. Each graphic represents one of the reference markers (**A**) or Immune Functional Assay (**B**) measured at D14. The first graphic column corresponds to REF set trajectory immunotypes, the second to mRNA set immunotypes, and the third to healthy volunteers. Immunotype’s D14 immune marker level is represented with violin plot, with the number of available samples at D14 indicated below each plot. To illustrate the differences in marker distribution between immunotypes of each set, we performed Wilcoxon tests and displayed horizontal bars between the concerned groups above the violins, with the p-value indicated above the bar. The tails of the violin plots were truncated to enhance the readability of the graphics. REF set: plasmatic IL6, plasmatic IL10, HLA-DR antibody/monocyte, T cells blood concentration, and percentage of immature neutrophils. mRNA set: normalized mRNA Cp in whole blood, IFNG, CD74, CX3CR1, IL7R, and IL1R2. HV: Healthy Volunteers. SEB: Staphylococcal Enterotoxin B. LPS: Lipopolysaccharide. D: Day. Immune Functional Assay: “IL2 SEB pg/mL”: release of IL2 measured after stimulation with SEB. “IFNg SEB pg/mL”: release of interferon gamma measured after stimulation with SEB. “TNFa LPS/NULL”: release of Tumor Necrosis Factor alpha after stimulation with LPS divided by the basal release of TNFα without stimulation (NULL)

When examining immune parameters at day 14, we observed a sustained immune dysregulation in Immunotype #1. This was characterized by diminished mHLA-DR levels, elevated percentage of immature neutrophils and elevated IL10 and IL6 levels, in contrast to both Immunotype #2 and to the range observed in healthy volunteers (Fig. 3A). Concurrently, we observed (Fig. 3B) a reduced immune function in Immunotype #1 relative to Immunotype #2 and the range observed in healthy volunteers. This was evidenced by an attenuated release of TNFα following stimulation with lipopolysaccharide (LPS), and a diminished release of IFNγ and IL2 after stimulation with Staphylococcal Enterotoxin B (SEB). These observations, in conjunction with the occurrence of HAI, suggest a degree of immunosuppression in Immunotype #1.

#### mRNA set biomarkers immunotypes

We examine the ability of mRNA set biomarkers to identify patients with altered immune system.

Here, the specific missing time points of the mRNA set biomarkers resulted in 326 patients’ trajectories with at least 2 time points out of 339 (Supplementary Fig. S5). This analysis conducted to the identification of two distinct immunotypes, according to PAC metric (see Supplementary Fig. S6B) using the same clustering algorithm as the one applied to the reference set markers. Immunotype #1 (n = 103), compared to Immunotype #2 (n = 223), had a significantly higher proportion of patients with complicated hospital courses (77% vs. 30%, Table 2). As for the immunotypes of the REF set, all secondary endpoints showed a significantly higher incidence in Immunotype #1 than in Immunotype #2. This included 30-day HAI (33% vs. 15%, sHR [CI] 2.56 [1.56; 4.21]), 30-day mortality (14% vs. 0%, sHR [CI] 22.68 [2.94; 175.02]), ICU stays > 7 days (52% vs. 21%), and 90-day mortality (18% vs. 5%), further emphasizing the association between Immunotype #1 and poorer clinical outcomes (see Fig. 1C, D). These results confirm the link between Immunotype #1 and worse clinical outcomes. Furthermore, these associations persisted even after controlling for clinical characteristics at admission, such as age, SOFA score, Charlson comorbidity index score, and initial injury, highlighting the relevance of such clustering (Supplementary Table S4). We also verified if the observed immunotypes could be fully explained by initial characteristics and found that the ability of classification into immunotypes by those characteristics was moderate, with an AUROC of 0.79 [0.73–0.83] (Supplementary Fig. S7B).

**Table 2:** mRNA set immunotypes clinical characterization

	mRNA set Immunotype #1 (n = 103)	mRNA set Immunotype #2 (n = 223)	*p*. Value
Baseline characteristics			
Category at admission			
Sepsis/Septic shock	59 (57%)	39 (18%)	** < 0.001**
Trauma	24 (23%)	107 (48%)	
Surgery	20 (19%)	77 (34%)	
Female gender	37 (36%)	77 (34%)	0.904
Age, years	65 [56–76]	56 [45–70]	** < 0.001**
Body mass index, kg/m^2^	25 [22–28]	25 [23–28]	0.939
Charlson score	2 [0–3]	1 [0–2]	**0.034**
Parameters at admission			
SAPS II score	44 [29–54]	24 [18–34]	** < 0.001**
SOFA score	8 [5–11]	3 [1–6]	** < 0.001**
Mechanical ventilation	74 (72%)	77 (34%)	** < 0.001**
Vasopressor use	81 (79%)	84 (38%)	** < 0.001**
D1 Lymphocyte	1.12 [0.76–1.68]	1.25 [0.87–1.85]	0.128
D1 NLR	10.53 [6.42–18.39]	8.21 [5.33–12.58]	**0.003**
Treatments during first week			
Hydrocortisone Hemisuccinate	22 (21.4%)	5 (2.2%)	** < 0.001**
Outcomes			
CHC (Complicated Hosp. Course)	79 (77%)	66 (30%)	** < 0.001**
D30 HAI	34 (33%)	33 (15%)	** < 0.001**
D30 Death	14 (14%)	1 (0%)	** < 0.001**
ICU LOS > 7D	50 (52%)	38 (21%)	** < 0.001**
D90 Death	18 (18%)	10 (5%)	** < 0.001**
D30 ICU free days	18 [8–23]	25 [22–27]	** < 0.001**
D30 Hosp. free days	2 [0–12]	17 [10–22]	** < 0.001**
D30 Mech. Vent. free days	26 [13–28]	29 [27–29]	** < 0.001**

SAPS II: Simplified Acute Physiological Score II. SOFA: Sequential Organ Failure Assessment score. D: Day. MV: mechanical ventilation. ICU: Intensive Care Unit. HAI: Healthcare Associated Infection. mRNA set: normalized mRNA Cp in whole blood, IFNg, CD74, CX3CR1, IL7R, and IL1R2. Data are presented as numbers and percentages (qualitative variables) and medians and 25th/75th percentiles (quantitative variables). Cohorts were compared either with analysis of variance (ANOVA) test in case of normally distributed data or with Kruskal Wallis test by ranks for continuous data, and Chi-squared test or Fisher’s exact test, where required, for categorical data. *p*. Values less than or equal to 0.05 were considered statistically significant and are highlighted in bold

Regarding biomarkers’ mean trajectory shape per immunotypes, Immunotype #1 exhibited a more altered immune response than Immunotype #2 during the first week after injury, as shown by the distance with the healthy volunteer distribution (Fig. 2). Specifically, IFNg, CD74, CX3CR1, and IL7R were less expressed than in healthy volunteers, while IL1R2 was more expressed. While Immunotype #2 mean marker trajectories converged towards the healthy volunteer Q1-Q3 range by the end of the first week, none of the Immunotype #1 mean marker trajectories reached it during the same period. While examining the reference markers measured at D14, patients in Immunotype #1 demonstrated significant differences in marker levels, further corroborating a delayed return to immune homeostasis compared to patients in Immunotype #2 (Fig. 3A). We also observed an altered immune function in the first Immunotype, as evidenced by TNFα release following stimulation with LPS, and IFNγ and IL2 release after stimulation with SEB (Fig. 3B). These observations suggest that patients in Immunotype #1 are more immunosuppressed than those in Immunotype #2.

Overall, these results suggest that the clustering based on mRNA set markers can also differentiate between distinct immunotypes, one highlighting more altered immunological characteristics and associated with poorer clinical outcomes.

### Comparison of immunotypes obtained with reference set markers and mRNA set markers

The comparison of immunotypes derived from the REF set markers and the mRNA set markers (Supplementary Fig. S8) showed a concordance of approximately 78%. A subset of 58 patients exhibited discordant immunotype classification, falling into mRNA immunotype #2 and REF set immunotype #1. These patients had better clinical outcomes and less persistent immune alterations at D14, in comparison to those concordantly classified in both REF set immunotype #1 and mRNA set immunotype #1 (Supplementary Table S5). On the contrary, a smaller subset of 12 patients, discordantly classified as mRNA set immunotype #1 and REF set immunotype #2, presented adverse clinical outcomes and more persistent immune alterations compared to those concordantly classified in both sets as immunotype #2 (Supplementary Table S6).

These findings indicate that despite utilizing different sets of markers, the two clustering methods are largely in agreement, thereby demonstrating a robust patient classification.

## Discussion

In this study, we characterized the immune response of critically ill patients using an unsupervised clustering method, KmL-3D, which allowed us to identify two distinct immunotypes based on the temporal evolution of multiple immune markers measured in whole blood samples. We found that one immunotype was associated with worse outcomes, such as increased risk of hospital-acquired infection and mortality, and prolonged hospital stay, while the other immunotype showed a faster and more favorable recovery. Our study provides a comprehensive understanding of the multiple facets and temporal evolution of the immune system of critically ill patients. Moreover, our study demonstrates the feasibility and interest of using a whole blood mRNA set, which can be easily measured using a single automated multiplexed PCR platform, to classify patients into immunotypes and potentially guide personalized therapies. Our results represent an important step toward precision medicine, a major aspect in the management of sepsis as reported in references [18, 28].

Our study is the first to use an unsupervised clustering method to identify immunotypes based on the temporal evolution of multiple immune markers in critically ill patients. It was applied in a consensus clustering framework, which allowed to limit bias in the number of groups identification and patient partitioning [6, 29, 30]. This method has several advantages over previous approaches that relied on a single immune biomarker-based clustering [6, 8, 31–34]. First, it allows to capture the complexity and heterogeneity of the immune response in critical illness, which is influenced by multiple factors, such as the nature and severity of the insult, the host characteristics (e.g. comorbidities, age …), and the timing of the measurement. Second, it enables us to identify immunotypes that are not only defined by the absolute levels of immune markers, but also by their dynamic changes over time, which may reflect different immune pathways and mechanisms. Third, it provides a data-driven and unbiased way to classify patients into immunotypes, without imposing assumptions or criteria that may not reflect the true nature of the immune response.

Building on this innovative method, our study further characterized the identified immunotypes, revealing distinct clinical outcomes and immune responses. Immunotype #1, characterized by a strong dysregulation of immune markers, including IL-6, IL-10, mHLA-DR, and immature neutrophils, indicated a state of concurrent inflammation and immunosuppression. Conversely, Immunotype #2 exhibited less dysregulation, suggesting a more balanced immune response. Immunotype #1 showed a persistence of its immunological dysregulation at day 14, while Immunotype #2 signs of immune recovery. The clinical implications of these immunotypes are significant. They are associated with different outcomes, such as the risk of hospital-acquired infection, mortality, and the duration of mechanical ventilation and hospital stay. This suggests that these immunotypes could serve as a valuable tool for stratifying patients according to their risk of adverse outcomes. The robustness of our findings was further corroborated by the clustering obtained with the mRNA set of biomarkers, which included genes related to innate and adaptive immunity, inflammation, and immunosuppression. This consistency across different sets of markers underscores the reliability of our immunotyping method. In addition, it’s worth noting that the mRNA set markers offer a practical advantage in a clinical setting. They can be quantified more easily at the bedside compared to traditional biomarkers, thanks to the use of a single automated multiplexed PCR platform. This ease of measurement could facilitate the clinical implementation of immunotype classification.

**Fig. 4:**
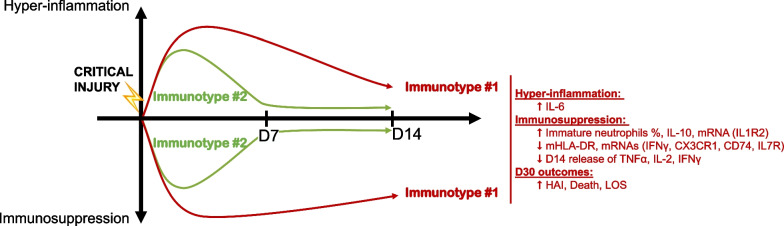
Empirical evidence supporting the theory of concurrent hyper-inflammation and immunosuppression. The theory of concurrent hyper-inflammation and immunosuppression has been discussed by numerous authors in literature related to critical injury [3, 10, 35–39].This theory proposes two distinct immune trajectories: one characterized by rapid immune recovery and another by delayed recovery, which is associated with an increased incidence of adverse outcomes. In this figure, we associate our empirical Immunotype #2 with the trajectory of rapid recovery (in green) and Immunotype #1 with the trajectory of delayed recovery (in red), thereby reinforcing the current theory

Our observations align with the theory presented in references [3, 10, 35, 36], suggesting that two distinct responses may be observed in critically ill patients: one characterized by a delayed recovery, linked to adverse outcomes, and another marked by a rapid immune system recovery leading to the patient’s prompt discharge. As such, the features of Immunotype #1 seem similar to the delayed immune recovery trajectory and the Immunotype #2 to the rapid recovery trajectory, further confirming this theory (Fig. 4).

Despite the promising results of our proof-of-concept study, there are several limitations that should be acknowledged and addressed in future research. Firstly, our clustering strategy was limited to the first week to minimize bias from missing data at later time points, which could occur if patients left the hospital or passed away. Possessing more comprehensive data at later time points could have allowed us to better define those immunotypes. Secondly, the immune markers were selected a priori, which may not fully capture the extent of immune dysregulation in critically ill patients. By choosing specific markers, we may have overlooked other relevant immune markers or pathways that could better characterize the immune system and offer potential drug targets and personalized immunomodulation. Future studies could explore the use of a more comprehensive panel of markers, such as cytokines, chemokines, cell subsets, mRNA, that could provide both insight on pathophysiology and potential drug targets. Thirdly, the mRNA markers have the advantage of being measurable in 1 h, which is beneficial for clinical settings. However, a limitation of our study is that we did not evaluate whether the mRNA set immunotypes could be discerned at a single time point, which might have more clinical utility than tracking the entire trajectory of patients. Future research should optimize the markers and investigate how they can inform treatment decisions.

## Conclusion

We applied an unsupervised clustering method to a panel of biomarkers to longitudinally profile the immune system of critically ill patients. This novel approach was the first to construct immunotypes based on the temporal dynamics of multiple immune biomarkers. We found two immunotypes that had distinct temporal patterns. One immunotype had persistent pro- and anti-inflammatory signals and worse outcomes, such as longer stays, higher mortality, and more infections and a persistent reduced response to ex vivo stimulation. Our study suggests that immunotyping based on temporal dynamics of immune markers could facilitate patient stratification. Further studies are needed to improve our immunotyping markers to better identify patient that could benefit of immunotherapy.

## Fundings

REALISM study received funding from the Agence Nationale de la Recherche through a grant awarded to BIOASTER (Grant No. #ANR-10-AIRT-03) and from bioMérieux, Sanofi and GSK. MB was supported by the Association Nationale de la Recherche et de la Technologie (ANRT), convention N° 2020/0371.

### Supplementary Information

**Supplementary material 1.**

## Abbreviations

AB/C: Antibody per cells
CHC: Complicated Hospital course
CI: Confidence interval
CX3CR1: C-X3-C motif chemokine receptor 1
D: Day
HAI: Healthcare-associated infection
HV: Healthy volunteer
ICU: Intensive care unit
IFNg: Interferon gamma
IL1R2: Interleukin 1 receptor type 2
IL2: Interleukine 2
IL6: Interleukin 6
IL7R: Interleukin 7 receptor
IL10: Interleukin 10
KmL3D: K-means for longitudinal data 3D
LPS: Lipopolysaccharide
mHLA-DR: Monocytic human leukocyte antigen-DR
mRNA: Messenger ribonucleic acid
mRNA set: Five mRNA measured in whole blood: IFNg, CD74, CX3CR1, IL7R, and IL1R2.
REF set: Five reference markers: plasmatic IL6, plasmatic IL10, monocytic HLA-DR antibody/cell, T cells blood concentration, and percentage of immature neutrophils.
ROC: Receiver operating characteristic
SAPS II: Simplified acute physiology score II
SEB: Staphylococcal enterotoxin B
sHR: Subdistribution hazard ratio
SOFA: Sequential Organ Failure Assessment
TNFa: Tumor necrosis factor alpha
TP: Time point
