# Supplementary material for: Integrated clustering of multiple immune marker trajectories reveals different immunotypes in severely injured patients
Source: Crit Care. 2024 Jul 15;28:240. doi: 10.1186/s13054-024-04990-4 (PMC11247757; doi:10.1186/s13054-024-04990-4)
Supplement: Supplementary file 1 — Supplementary material 1. [file 13054_2024_4990_MOESM1_ESM.docx]

# Supplementary data

Supplementary Methods

Supplementary method 1: Immunotypes Definition through unsupervised trajectory clustering

Please refer to Supplementary figure 4 for graphical illustration.

In this study, we utilized an unsupervised clustering method to group multiple marker trajectories simultaneously (markers co-evolution over time) of patients according to the similarities in their shapes. The resulting groups were referred to as "immunotypes" and are thus constituted of patients whose immune response exhibited similar behavior as determined by the clustering algorithm. The grouping was based exclusively on the marker trajectories without any additional information. Immunotypes were labeled from 1 to n according to decreasing CHC outcome.

Given the absence of prior assumptions regarding the shape of marker trajectories, we opted for a non-parametric and flexible method. To cluster multiple marker trajectories simultaneously, taking into account their co-evolution, we employed the KmL-3D - K-means for Longitudinal data R package 2.4.6 [1, 2]. The implementation of the KmL-3D package followed a two-step process. In the first step, the markers were centered and scaled to avoid bias from different scales across multiple markers. Subsequently, the k-means algorithm with a Gower adjusted Euclidean distance metric was applied to cluster marker trajectories. This metric is designed to handle missing data and accommodate the multi-trajectory setting, as extensively detailed in [3]. In the second step, linear interpolation was employed to impute missing values within each cluster. This approach aimed to ensure that the imputed values conformed to the population mean trajectory shape before calculating the Calinski-Harabasz metric. This metric assesses the quality of partitioning by representing the ratio between within-cluster and between-cluster dispersion. Because k-means methods can produce suboptimal partitioning results due to its reliance on random initialization we executed KmL-3D 7,000 times and chose the run with the highest Calinski-Harabasz metric, ensuring we had attained the most optimal partitioning.

### Number of immunotypes

We used the clustering method to derive 2 to 6 immunotypes. Our assumption was that there are at least 2 types of immune responses existing in patients, and that more than 6 would result in over-partitioning. The empirically correct number of immunotypes was determined after clusters were determined, based on the stability of patient grouping, as described in the following section “Control of the risk of overfitting for determining number of immunotypes and partitioning”.

### Control of the risk of overfitting for determining the number of immunotypes and partitioning

#### Bootstrap

To address the risk of overfitting when employing a clustering method on a single dataset, which may lead to biased estimations of group numbers and/or partitioning, we carried out bootstrapping. By generating a hundred datasets based on the REALISM dataset, referred to as "bootstrapped datasets" or "BsD", we allowed for varying distributions of patient characteristic in each resulting BsD. Clustering was then applied to each BsD, with one for each pre-determined number of immunotypes.

#### Summary of 100-BsD partitioning

A summary of the 100-BsD partitioning was achieved using the consensus clustering methodology [4] for each number of immunotypes. This method constructs a consensus matrix, which is a frequency matrix representing the pairwise immunotype agreement for patients' trajectories across all clustered bootstrapped datasets. The consensus matrix captures how often each pair of patients' trajectories is assigned to the same immunotype. A frequency value of 0 means that the two patients’ trajectories were never clustered in the same immunotype over all BsD, while a frequency of 1 means that the two patients’ trajectories were always clustered in the same immunotype.

#### Determining the number of immunotypes.

The most stable clustering is the one with the lowest number of pairs having a frequency between 0.1 and 0.9, i.e. the lowest number of paired patients’ trajectories that are inconsistently grouped together. This stability is quantified by a statistic called proportion of ambiguous clustering (PAC) [4]. The PAC represents the fraction of pairs with an immunotype match frequency between 0.1 and 0.9 (the "ambiguous clustering" range). We identified the optimal number of immunotypes for a given clustering configuration by minimizing this metric across all consensus matrices.

#### Determination of immunotypes

The consensus matrix of the retained number of groups was clustered using hierarchical clustering with an Euclidean distance and Ward’s linkage method [5]. The resulting tree was cut according to the previously selected number of groups. Immunotypes were then numbered in decreasing order of their enrichment in deleterious clinical outcomes, as measured by CHC.

### Typical trajectory of immunotypes

As the non-parametric clustering methods used to define immunotypes can identify non-linear shapes, we used the LOESS (Locally Weighted Scatterplot Smoothing) [6] non-parametric method to smooth marker measurements within each immunotype. The resulting smoothed curves define the mean evolution of each immunotype.

REFERENCES

1. Genolini, C., et al., *KmL3D: a non-parametric algorithm for clustering joint trajectories.* Comput Methods Programs Biomed, 2013. **109**(1): p. 104-11.

2. Genolini, C., X. Alacoque, M. Sentenac, and C. Arnaud, *kml and kml3d: R Packages to Cluster Longitudinal Data.* Journal of Statistical Software, 2015. **65**(4): p. 1 - 34.

3. Genolini, C. and B. Falissard, *KmL: k-means for longitudinal data.* Computational Statistics, 2009. **25**(2): p. 317-328.

4. Șenbabaoğlu, Y., G. Michailidis, and J.Z. Li, *Critical limitations of consensus clustering in class discovery.* Scientific Reports, 2014. **4**(1): p. 6207.

5. Kiselev, V.Y., et al., *SC3: consensus clustering of single-cell RNA-seq data.* Nat Methods, 2017. **14**(5): p. 483-486.

6. Cleveland, W.S., *Robust Locally Weighted Regression and Smoothing Scatterplots.* Journal of the American Statistical Association, 1979. **74**(368): p. 829-836.

Supplementary Figures

Supplementary figure 1: REFset markers spearman correlation at different time point

The figure consists of three panels (A-C), each showing the Spearman correlations for five REFset markers at different post-injury time frames. Panel A shows 1-2 days, Panel B shows 3-4 days, and Panel C shows 5-7 days post-injury. Each panel features a matrix plot: density plots on the diagonal, Spearman coefficients with significance levels in the upper triangle (0.05-0.01*; 0.01-0.001**, <0.001***), and scatter plots of marker pairs in the lower triangle.


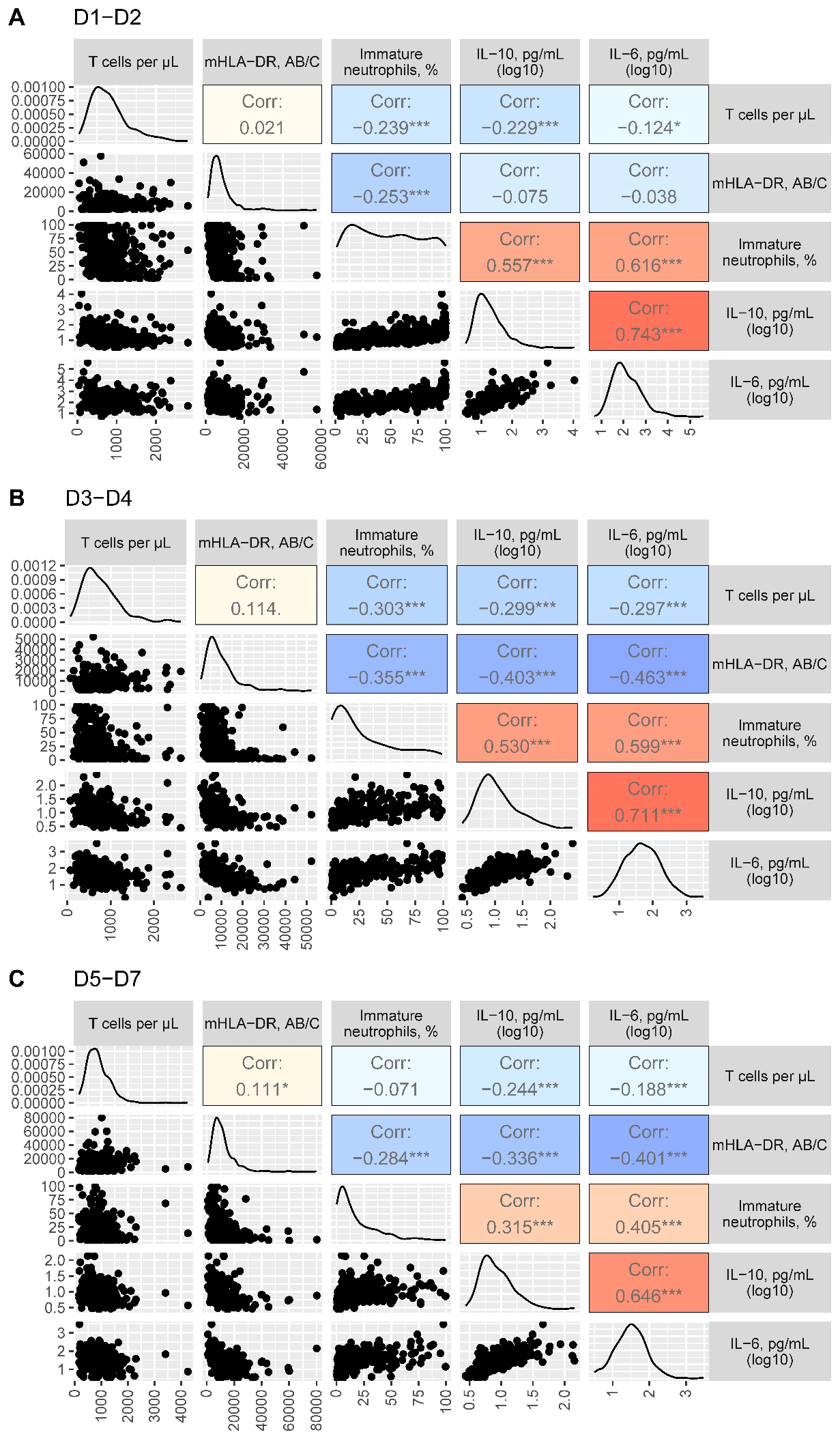


Supplementary figure 2: mRNAset markers spearman correlation

T he figure consists of three panels (A-C), each showing the Spearman correlations for five REFset markers at different post-injury time frames. Panel A shows 1-2 days, Panel B shows 3-4 days, and Panel C shows 5-7 days post-injury. Each panel features a matrix plot: density plots on the diagonal, Spearman coefficients with significance levels in the upper triangle (0.05-0.01*; 0.01-0.001**, <0.001***), and scatter plots of marker pairs in the lower triangle.


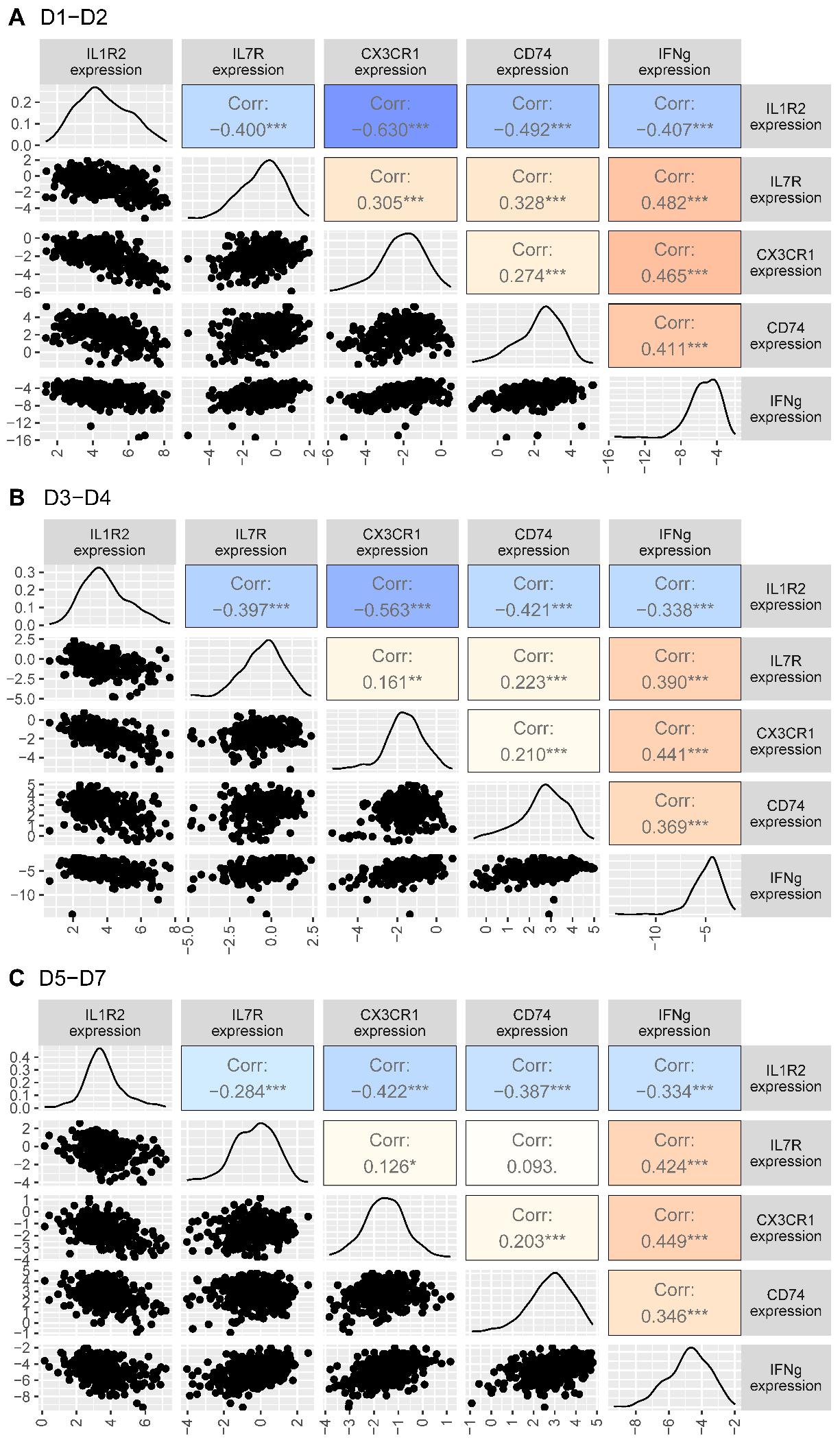


Supplementary figure 3: Study workflow

This study focused on the longitudinal characterization of the immune system in critically ill patients from the REALISM cohort, which includes 339 patients with at least two time points during the first week after study admission (top graphic). These time points were analyzed for both 5 "reference" biomarkers (REF set, light blue square on the left) and 5 mRNA biomarkers (mRNA set, blue square on the right). Each set was independently clustered, considering 2 to 6 clusters. The resulting clusters of homogeneous marker co-evolution were termed "Immunotypes". Each Immunotype was then characterized by outcome incidence, and the immunotypes classification from REF set clustering were compared to those from mRNA set clustering.


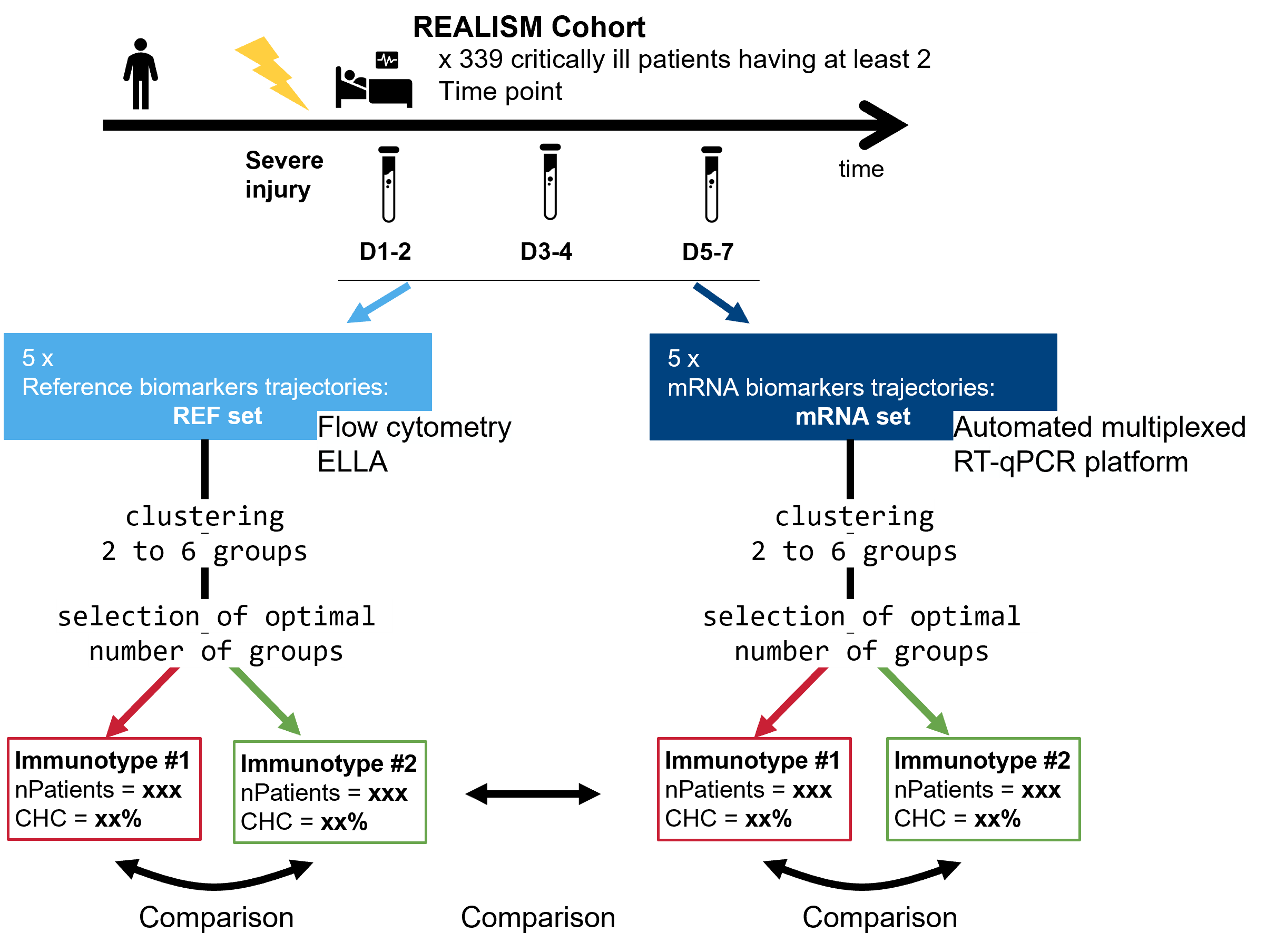


Supplementary figure 4: Immunotyping method workflow.


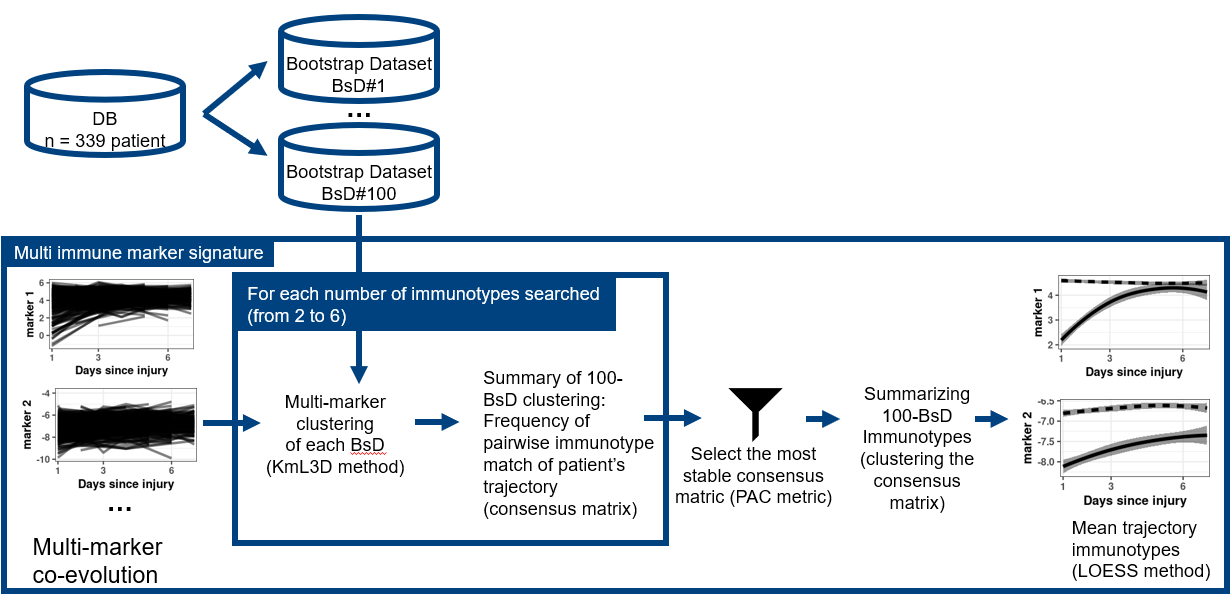
This figure is an illustration of the supplementary methods detailing how we identify immunotypes.

Supplementary figure 5: Time point exclusion flowchart: REF set and mRNA set.


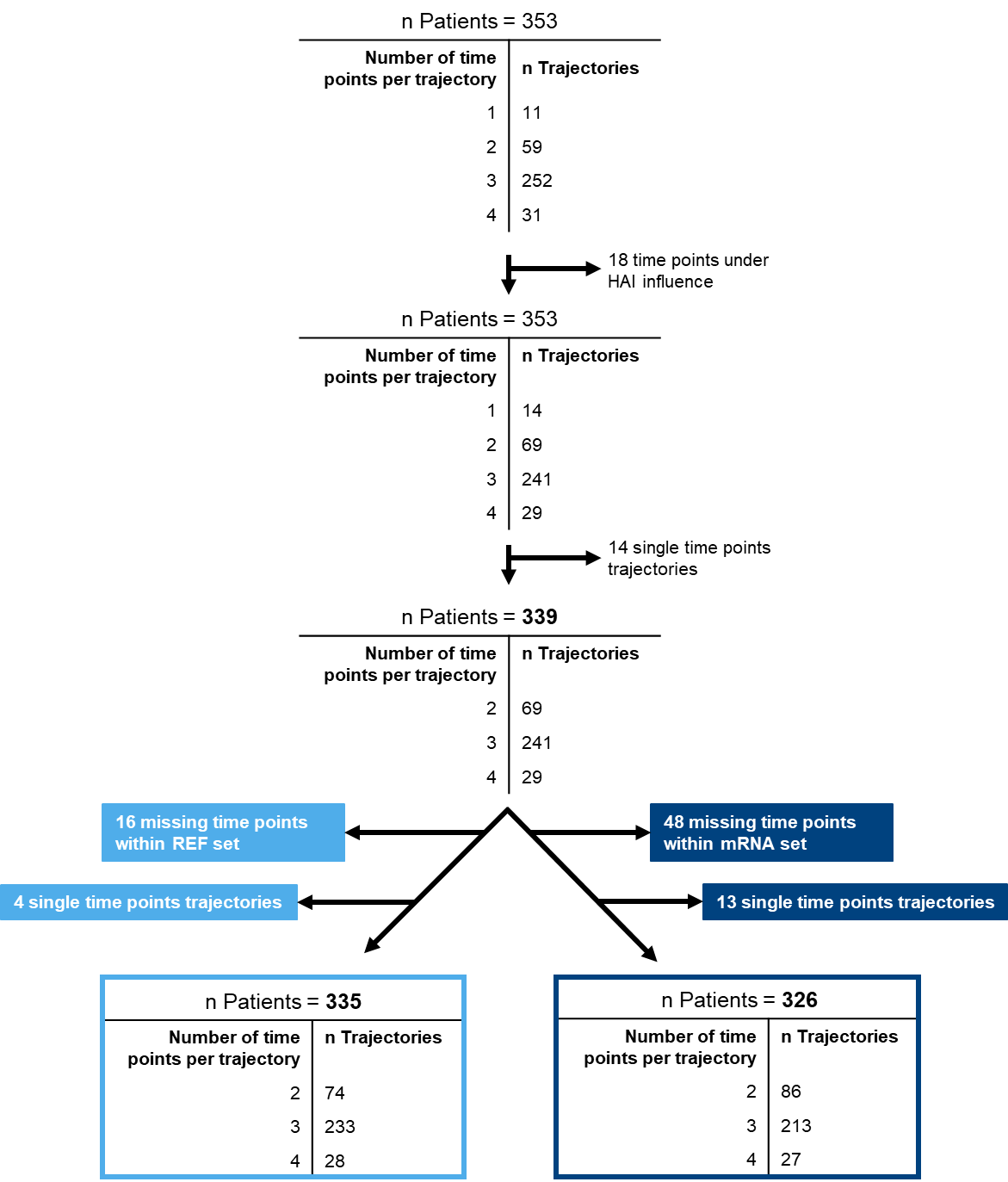
One table is presented for each stage of the flow chart, with the table header indicating the total number of patients. Below the header are two columns representing the number of time points per trajectory (left) and the corresponding count of trajectories (right). The first exclusion step corresponds to the removal of time points occurring after HAI to avoid potential bias in immune marker measurement levels. The second exclusion step corresponds to the removal of single time points. The resulting analyzed subcohort corresponds to missing time point removal specific to the measured REF set biomarkers (left) and the removal of single time points, which is also applied to the mRNA set biomarkers (right). REF set: plasmatic IL6, plasmatic IL10, monocytic HLA-DR antibody / cell, T cells blood concentration, and percentage of immature neutrophils; mRNA set: normalized mRNA Cp in whole blood, IFNg, CD74, CX3CR1, IL7R, and IL1R2; HAI: Healthcare-Associated Infection.

Supplementary figure 6: Optimal number of immunotypes per clustering.


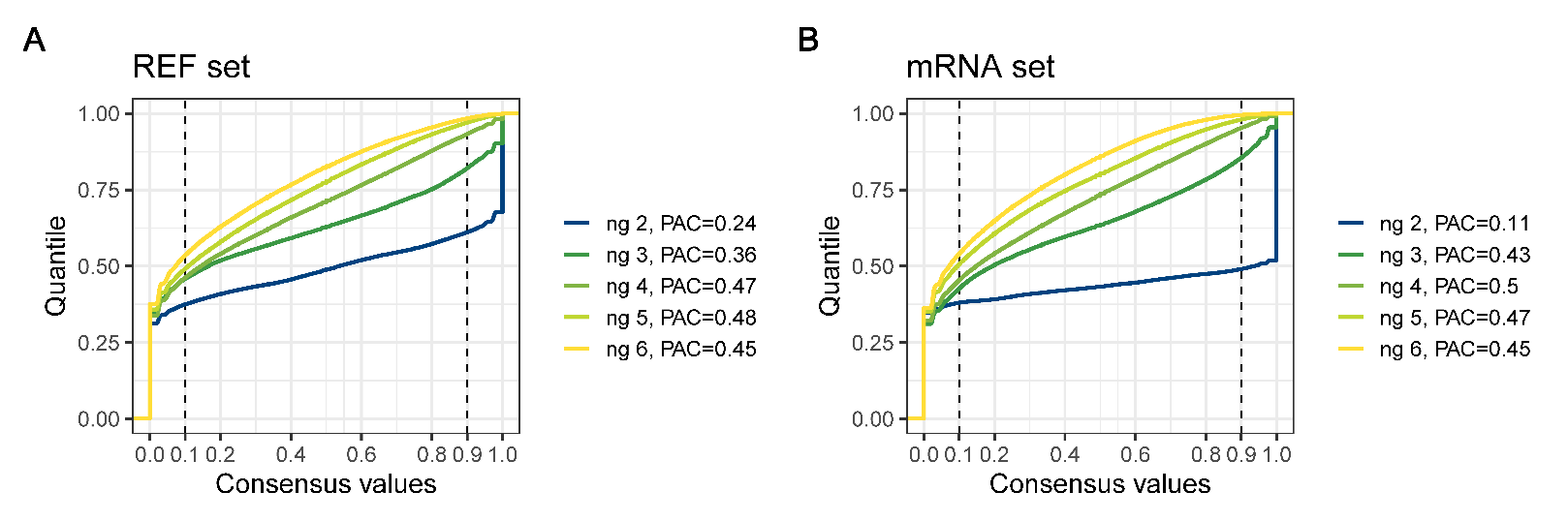
We derived the cumulative distribution function from the consensus clustering matrices for each number of groups (or immunotypes) searched for the REF set (A) and the mRNA set (B). The legend reports the PAC metric, and the lowest PAC indicates the most stable clustering assumed to be the optimal number of immunotypes. REF set: plasmatic IL6, plasmatic IL10, monocytic HLA-DR antibody / cell, T cells blood concentration, and percentage of immature neutrophils; mRNA set: normalized mRNA Cp in whole blood, IFNg, CD74, CX3CR1, IL7R, and IL1R2; ng: number of groups; PAC: Proportion of Ambiguous Clusters.

Supplementary figure 7: Predictive Performance of Logistic Regression Models for Immunotype Prediction Based on Initial Patient Characteristics.

Logistic regression analysis, employing stepwise variable selection steered by the Akaike Information Criterion (AIC). The analysis was conducted on two dependent variable: REFset immunoytpes (A) and mRNAset immunotypes(B). The models were developed using 30 repeated 6-fold cross-validations to predict immunotypes, considering initial patient characteristics such as Age, SOFA score on Day 1, Charlson Comorbidity Index Score, and initial injury (grouped into Sepsis, Trauma, or Surgery).

Although not depicted in this figure, the REFset cohort identified Age and SOFA_D1 as significant predictors post variable selection. In contrast, the mRNAset cohort recognized SOFA_D1 and Trauma (compared to the sepsis reference) as significant predictors.


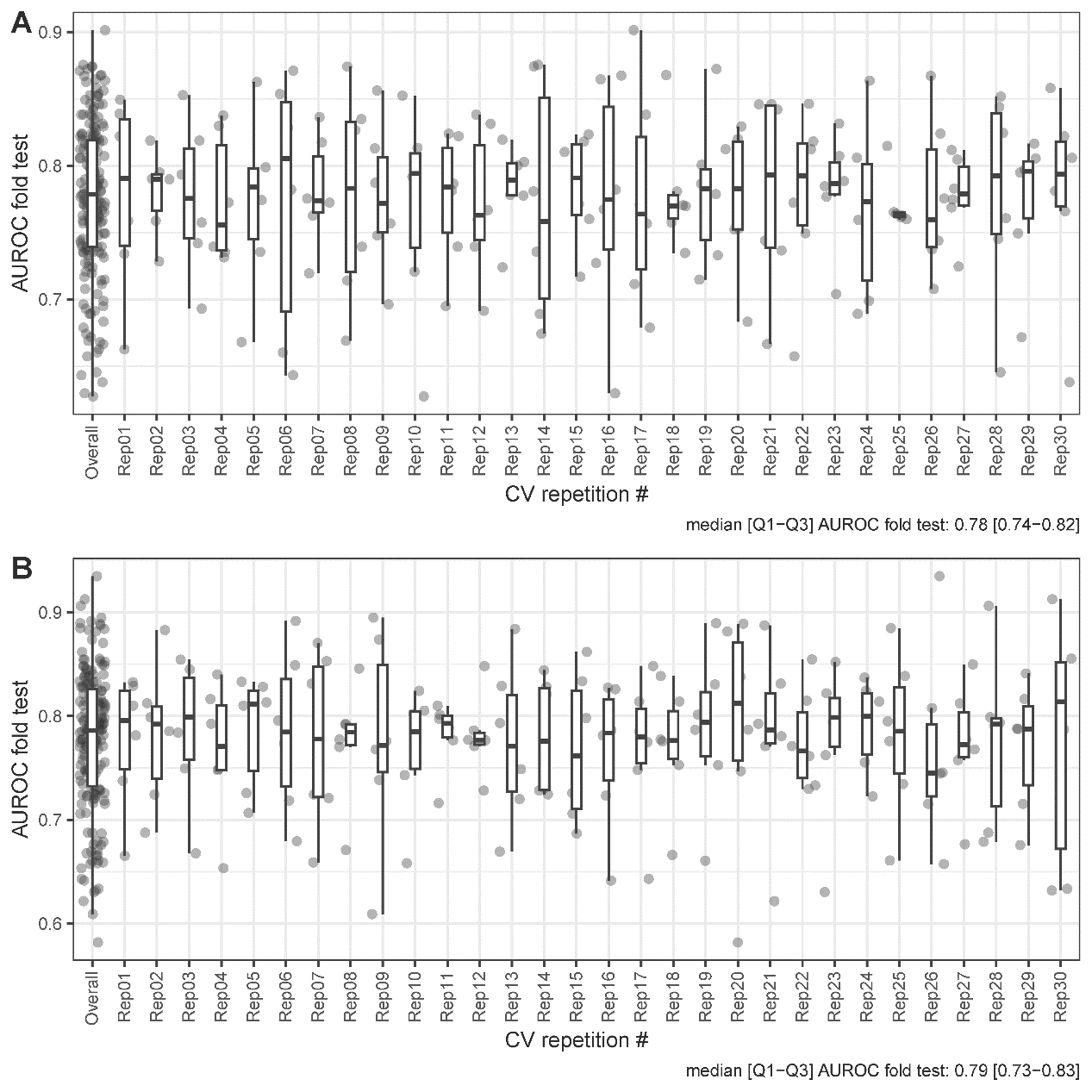
The boxplots illustrate the distribution of Area Under the Receiver Operating Characteristic (AUROC) scores across all test folds in the cross-validation. The leftmost boxplot aggregates all 30 repetitions, while the subsequent boxplots to the right represent the AUROC distribution for each 6-fold test within every repetition. The median AUROC [Q1-Q3] for all repetitions and fold tests is displayed beneath each graph.

Supplementary figure 8: REF set immunotypes and mRNA set immunotypes patient flows.

Sankey plot illustrating the patient flow between REF set immunotypes #1 (red, left) and #2 (green, left), and mRNA set immunotypes #1 (red, right) and #2 (green, right). Flows are depicted as colored bands, representing the REF set immunotype colors, with the number of patients in each flow indicated on the bands.


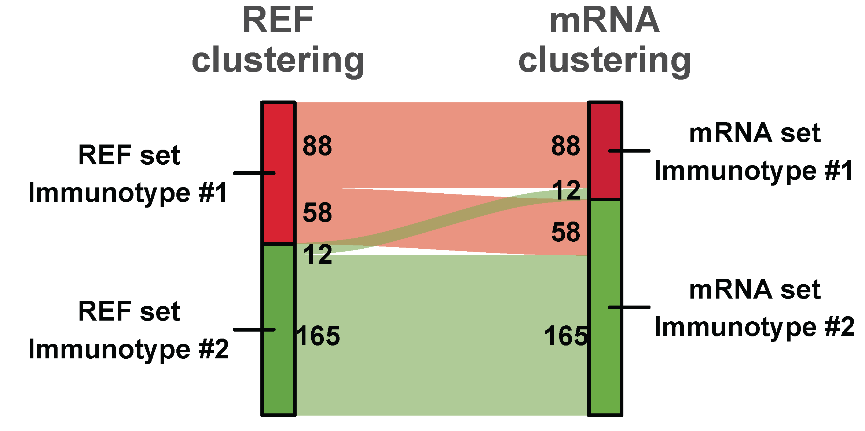


Supplementary Tables

Supplementary table 1: Global cohort characteristics.

SAPS II: Simplified Acute Physiological Score II. SOFA: Sequential Organ Failure Assessment score. D: Day. MV: mechanical ventilation. ICU: Intensive Care Unit. HAI: Healthcare Associated Infection. Data are presented as numbers and percentages (qualitative variables) and medians and 25th/75th percentiles (quantitative variables).

|  | **REALISM cohort (n=339)** |
| --- | --- |
| **Baseline characteristics** |  |
| **Category at admission** |  |
| Sepsis/Septic shock | 102 (30%) |
| Trauma | 101 (30%) |
| Surgery | 136 (40%) |
| **Female gender** | 117 (34%) |
| **Age, years** | 60 [47-71] |
| **Body mass index, kg/m²** | 25 [22-28] |
| **Charlson score** | 1 [0-2] |
|  |  |
| **Parameters at admission** |  |
| **SAPS II score** | 29 [20-43] |
| **SOFA score** | 5 [1-8] |
| **Mechanical ventilation** | 157 (46%) |
| **Vasopressor use** | 172 (51%) |
|  |  |
| **Outcomes** |  |
| **CHC (Complicated Hosp. Course)** | 151 (44%) |
| D30 HAI | 71 (21%) |
| D30 Death | 16 (5%) |
| ICU LOS > 7D | 90 (31%) |
| **D90 Death** | 29 (9%) |
| **D30 ICU free days** | 24 [19-26] |
| **D30 Hosp. free days** | 15 [2-21] |
| **D30 Mech. Vent. free days** | 28 [24-29] |
|  |  |

Supplementary table 2: Complicated Hospital Course – CHC – outcome clinical characteristics

|  | **CHC (n=151)** | **No CHC (n=188)** | **p. value** |
| --- | --- | --- | --- |
| **Baseline characteristics** |  |  |  |
| **Category at admission** |  |  |  |
| Sepsis/Septic shock | 61 (40%) | 41 (22%) | **<0.001** |
| Trauma | 56 (37%) | 80 (43%) |  |
| Surgery | 34 (22%) | 67 (36%) |  |
| **Female gender** | 52 (34%) | 65 (35%) | 1.000 |
| **Age, years** | 63 [52-73] | 56 [44-69] | **0.002** |
| **Body mass index, kg/m²** | 26 [22-30] | 24 [22-27] | **0.017** |
| **Charlson score** | 1 [0-3] | 1 [0-2] | 0.130 |
|  |  |  |  |
| **Parameters at admission** |  |  |  |
| **SAPS II score** | 35 [26-49] | 23 [17-35] | **<0.001** |
| **SOFA score** | 7 [2-10] | 3 [1-6] | **<0.001** |
| **Mechanical ventilation** | 99 (66%) | 58 (31%) | **<0.001** |
| **Vasopressor use** | 100 (66%) | 72 (38%) | **<0.001** |
|  |  |  |  |
| **Outcomes** |  |  |  |
| **D90 Death** | 26 (17%) | 3 (2%) | **<0.001** |
| **D30 ICU free days** | 18 [10-22] | 26 [24-27] | **<0.001** |
| **D30 Hosp. free days** | 3 [0-12] | 18 [12-23] | **<0.001** |
| **D30 Mech. Vent. free days** | 26 [12-29] | 29 [28-29] | **<0.001** |

SAPS II: Simplified Acute Physiological Score II. SOFA: Sequential Organ Failure Assessment score. D: Day. MV: mechanical ventilation. ICU: Intensive Care Unit. HAI: Healthcare Associated Infection. Data are presented as numbers and percentages (qualitative variables) and medians and 25th/75th percentiles (quantitative variables). Cohorts were compared either with analysis of variance (ANOVA) test in case of normally distributed data or with Kruskal Wallis test by ranks for continuous data, and Chi-squared test or Fisher’s exact test, where required, for categorical data.

Supplementary table 3: REF set immunotypes outcomes association, adjusted for confounders.

Results of the competing risks regression (CRR) analysis using the Fine and Gray model and REF set immunotypes adjusted for age, Sequential Organ Failure Assessment score on day 1 (SOFA_D1), Charlson Comorbidity Index score (Charlson_Score), and initial injury subgroups (sepsis, trauma, or surgery). The outcomes of interest are healthcare associated infection (HAI), death, and hospital discharge. The subdistribution hazard ratio (sHR) and the 95% confidence interval (CI) are reported for each outcome and immunotype. A sHR > 1 indicates a higher risk or probability of the outcome, while a sHR < 1 indicates a lower risk or probability of the outcome.

|  |  |  | **CRR: HAI** | | |  | **CRR: Death** | | |  | **CRR: Hosp. discharge** | | |
| --- | --- | --- | --- | --- | --- | --- | --- | --- | --- | --- | --- | --- | --- |
|  | **Total** |  | **Events** | **sHR (95% CI)** | **p** |  | **Events** | **sHR (95% CI)** | **p** |  | **Events** | **sHR (95% CI)** | **p** |
|  | **n = 335 (%)** |  | **n = 64 (19)** |  |  |  | **n = 10 (3)** |  |  |  | **n = 224 (67)** |  |  |
| **Age** | | | | | | | | | | | | | |
| 1 yr incr. | 335 (100) |  | 64 (100) | 1.02 (1.00, 1.03) | 0.029 |  | 10 (100) | 1.05 (1.00, 1.10) | 0.076 |  | 224 (100) | 0.99 (0.98, 1.00) | 0.025 |
| **SOFA_D1** | | | | | | | | | | | | | |
| 1 point incr. | 335 (100) |  | 64 (100) | 1.05 (0.97, 1.14) | 0.22 |  | 10 (100) | 1.16 (0.98, 1.38) | 0.085 |  | 224 (100) | 0.95 (0.91, 1.00) | 0.035 |
| **Charlson_Score** | | | | | | | | | | | | | |
| 1 point incr. | 335 (100) |  | 64 (100) | 1.02 (0.89, 1.18) | 0.77 |  | 10 (100) | 1.11 (0.91, 1.34) | 0.31 |  | 224 (100) | 0.92 (0.83, 1.02) | 0.11 |
| **Subgroup** | | | | | | | | | | | | | |
| Sepsis | 99 (30) |  | 19 (30) |  | - |  | 9 (90) |  | - |  | 48 (21) |  | - |
| Trauma | 136 (41) |  | 22 (34) | 2.44 (1.10, 5.40) | 0.028 |  | 1 (10) | 0.68 (0.05, 9.00) | 0.77 |  | 103 (46) | 0.93 (0.59, 1.48) | 0.77 |
| Surgery | 100 (30) |  | 23 (36) | 3.11 (1.40, 6.92) | 0.0054 |  | 0 (0) | 0.00 (0.00, 0.00) | < 0.001 |  | 73 (33) | 1.19 (0.75, 1.90) | 0.46 |
| **Immunotype** | | | | | | | | | | | | | |
| REF_set_Immunotype_2 | 184 (55) |  | 18 (28) |  | - |  | 0 (0) |  | - |  | 158 (71) |  | - |
| REF_set_Immunotype_1 | 151 (45) |  | 46 (72) | 3.60 (1.98, 6.57) | < 0.001 |  | 10 (100) | 1470969.55 (632740.48, 3419650.67) | < 0.001 |  | 66 (29) | 0.36 (0.26, 0.48) | < 0.001 |

Supplementary table 4: mRNA set immunotypes outcomes association, adjusted for confounders

Results of the competing risks regression (CRR) analysis using the Fine and Gray model and mRNA set immunotypes adjusted for age, Sequential Organ Failure Assessment score on day 1 (SOFA_D1), Charlson Comorbidity Index score (Charlson_Score), and initial injury subgroups (sepsis, trauma, or surgery). The outcomes of interest are healthcare associated infection (HAI), death, and hospital discharge. The subdistribution hazard ratio (sHR) and the 95% confidence interval (CI) are reported for each outcome and immunotype. A sHR > 1 indicates a higher risk or probability of the outcome, while a sHR < 1 indicates a lower risk or probability of the outcome.

|  |  |  | **CRR: HAI** | | |  | **CRR: Death** | | |  | **CRR: Hosp. discharge** | | |
| --- | --- | --- | --- | --- | --- | --- | --- | --- | --- | --- | --- | --- | --- |
|  | **Total** |  | **Events** | **sHR (95% CI)** | **p** |  | **Events** | **sHR (95% CI)** | **p** |  | **Events** | **sHR (95% CI)** | **p** |
|  | **n = 326 (%)** |  | **n = 61 (19)** |  |  |  | **n = 11 (3)** |  |  |  | **n = 219 (67)** |  |  |
| **Age** | | | | | | | | | | | | | |
| 1 yr incr. | 326 (100) |  | 61 (100) | 1.02 (1.00, 1.03) | 0.051 |  | 11 (100) | 1.07 (1.03, 1.10) | < 0.001 |  | 219 (100) | 0.99 (0.98, 0.99) | < 0.001 |
| **SOFA_D1** | | | | | | | | | | | | | |
| 1 point incr. | 326 (100) |  | 61 (100) | 1.07 (0.97, 1.17) | 0.17 |  | 11 (100) | 1.14 (0.96, 1.35) | 0.12 |  | 219 (100) | 0.91 (0.87, 0.96) | < 0.001 |
| **Charlson_Score** | | | | | | | | | | | | | |
| 1 point incr. | 326 (100) |  | 61 (100) | 1.04 (0.91, 1.19) | 0.59 |  | 11 (100) | 1.05 (0.85, 1.29) | 0.65 |  | 219 (100) | 0.93 (0.84, 1.03) | 0.14 |
| **Subgroup** | | | | | | | | | | | | | |
| Sepsis | 98 (30) |  | 19 (31) |  | - |  | 10 (91) |  | - |  | 47 (21) |  | - |
| Trauma | 131 (40) |  | 20 (33) | 2.41 (1.04, 5.63) | 0.041 |  | 1 (9) | 0.69 (0.05, 10.37) | 0.79 |  | 100 (46) | 0.81 (0.51, 1.27) | 0.35 |
| Surgery | 97 (30) |  | 22 (36) | 3.08 (1.27, 7.48) | 0.013 |  | 0 (0) | 0.00 (0.00, 0.00) | < 0.001 |  | 72 (33) | 1.10 (0.70, 1.74) | 0.67 |
| **Immunotype** | | | | | | | | | | | | | |
| mRNA_set_Immunotype_2 | 223 (68) |  | 29 (48) |  | - |  | 1 (9) |  | - |  | 179 (82) |  | - |
| mRNA_set_Immunotype_1 | 103 (32) |  | 32 (52) | 2.59 (1.35, 4.96) | 0.0041 |  | 10 (91) | 5.57 (0.60, 51.59) | 0.13 |  | 40 (18) | 0.39 (0.28, 0.56) | < 0.001 |

Supplementary table 5: Clinical characteristics of patients from REF set Immunotype #1 according to their mRNA set immunotype classification

SAPS II: Simplified Acute Physiological Score II. SOFA: Sequential Organ Failure Assessment score. D: Day. MV: mechanical ventilation. ICU: Intensive Care Unit. HAI: Healthcare Associated Infection. Data are presented as numbers and percentages (qualitative variables) and medians and 25th/75th percentiles (quantitative variables). Cohorts were compared either with analysis of variance (ANOVA) test in case of normally distributed data or with Kruskal Wallis test by ranks for continuous data, and Chi-squared test or Fisher’s exact test, where required, for categorical data.

|  | **REFset #1  to mRNAset #1 (n=88)** | **REFset #1  to mRNAset #2 (n=58)** | **p. value** |
| --- | --- | --- | --- |
| **Baseline characteristics** |  |  |  |
| **Category at admission** |  |  |  |
| Sepsis/Septic shock | 52 (59%) | 19 (33%) | **0.004** |
| Trauma | 19 (22%) | 26 (45%) |  |
| Surgery | 17 (19%) | 13 (22%) |  |
| **Female gender** | 31 (35%) | 13 (22%) | 0.142 |
| **Age, years** | 64 [56-76] | 57 [44-69] | **0.006** |
| **Body mass index, kg/m²** | 25 [22-29] | 25 [23-28] | 0.946 |
| **Charlson score** | 2 [0-2] | 1 [0-3] | 0.430 |
|  |  |  |  |
| **Parameters at admission** |  |  |  |
| **SAPS II score** | 45 [30-54] | 31 [22-38] | **<0.001** |
| **SOFA score** | 8 [5-11] | 6 [3-8] | **<0.001** |
| **Mechanical ventilation** | 67 (76%) | 32 (55%) | **0.013** |
| **Vasopressor use** | 72 (82%) | 36 (62%) | **0.014** |
|  |  |  |  |
| **Outcomes** |  |  |  |
| **CHC (Complicated Hosp. Course)** | 70 (80%) | 26 (45%) | **<0.001** |
| D30 HAI | 31 (35%) | 15 (26%) | 0.313 |
| D30 Death | 11 (13%) | 1 (2%) | **0.028** |
| ICU LOS > 7D | 46 (57%) | 18 (33%) | **0.012** |
| **D90 Death** | 14 (16%) | 5 (9%) | 0.309 |
| **D30 ICU free days** | 17 [7-22] | 24 [20-26] | **<0.001** |
| **D30 Hosp. free days** | 0 [0-12] | 10 [0-16] | **0.001** |
| **D30 Urin. Cat. free days** | 18 [0-25] | 25 [19-27] | **<0.001** |
| **D30 Venous Cat. free days** | 8 [0-21] | 24 [10-26] | **<0.001** |

Supplementary table 6: Clinical characteristics of patients from REF set Immunotype #2 according to their mRNA set Immunotype classification

SAPS II: Simplified Acute Physiological Score II. SOFA: Sequential Organ Failure Assessment score. D: Day. MV: mechanical ventilation. ICU: Intensive Care Unit. HAI: Healthcare Associated Infection. Data are presented as numbers and percentages (qualitative variables) and medians and 25th/75th percentiles (quantitative variables). Cohorts were compared either with analysis of variance (ANOVA) test in case of normally distributed data or with Kruskal Wallis test by ranks for continuous data, and Chi-squared test or Fisher’s exact test, where required, for categorical data.

|  | **REFset #2  to mRNAset #2 (n=165)** | **REFset #2  to mRNAset #1 (n=12)** | **p. value** |
| --- | --- | --- | --- |
| **Baseline characteristics** |  |  |  |
| **Category at admission** |  |  |  |
| Sepsis/Septic shock | 20 (12%) | 4 (33%) | 0.127 |
| Trauma | 81 (49%) | 5 (42%) |  |
| Surgery | 64 (39%) | 3 (25%) |  |
| **Female gender** | 64 (39%) | 4 (33%) | 0.770 |
| **Age, years** | 56 [45-70] | 68 [23-75] | 0.523 |
| **Body mass index, kg/m²** | 25 [23-28] | 25 [19-27] | 0.438 |
| **Charlson score** | 1 [0-2] | 2 [0-3] | 0.304 |
|  |  |  |  |
| **Parameters at admission** |  |  |  |
| **SAPS II score** | 22 [15-32] | 34 [19-46] | 0.091 |
| **SOFA score** | 2 [1-5] | 4 [1-8] | 0.115 |
| **Mechanical ventilation** | 45 (27%) | 5 (42%) | 0.323 |
| **Vasopressor use** | 48 (29%) | 6 (50%) | 0.191 |
|  |  |  |  |
| **Outcomes** |  |  |  |
| **CHC (Complicated Hosp. Course)** | 40 (24%) | 6 (50%) | 0.081 |
| D30 HAI | 18 (11%) | 2 (17%) | 0.629 |
| D30 Death | 0 (0%) | 1 (8%) | 0.068 |
| ICU LOS > 7D | 20 (15%) | 2 (17%) | 1.000 |
| **D90 Death** | 5 (3%) | 1 (8%) | 0.356 |
| **D30 ICU free days** | 26 [23-27] | 25 [22-27] | 0.819 |
| **D30 Hosp. free days** | 19 [13-23] | 10 [5-16] | **0.003** |
| **D30 Urin. Cat. free days** | 27 [24-28] | 23 [20-26] | 0.056 |
| **D30 Venous Cat. free days** | 23 [18-27] | 23 [18-26] | 0.830 |
